# Effect of Aging on Chemical and Rheological Properties of Bitumen

**DOI:** 10.3390/polym10121345

**Published:** 2018-12-05

**Authors:** Zhen Yang, Xiaoning Zhang, Zeyu Zhang, Bingjie Zou, Zihan Zhu, Guoyang Lu, Wei Xu, Jiangmiao Yu, Huayang Yu

**Affiliations:** 1 School of Civil Engineering and Transportation, South China University of Technology, Wushan Road, Tianhe District, Guangzhou 510000, China; 201410101329@mail.scut.edu.cn (Z.Y.); ctxnzh@scut.edu.cn (X.Z.); maytwentyz1118@163.com (B.Z.); 201730187102@mail.scut.edu.cn (Z.Z.); xuweib@scut.edu.cn (W.X.); yujm@scut.edu.cn (J.Y.); 2 Institute of Highway Engineering, RWTH Aachen University, Mies-van-der-Rohe-Street 1, 52074 Aachen, Germany; zeyu.zhang@isac.rwth-aachen.de (Z.Z.); lu@isac.rwth-aachen.de (G.L.); 3 Anhui Communications Vocational & Technical College, Taihu East Road, Baohe District, Hefei 230051, China

**Keywords:** aging, functional group, micro mechanics, rheological property, bitumen

## Abstract

Engineering performance of asphalt pavement highly depends on the properties of bitumen, the bonding material to glue aggregates and fillers together. During the service period, bitumen is exposed to sunlight, oxygen and vehicle loading which in turn leads to aging and degradation. A comprehensive understanding of the aging mechanism of bitumen is of critical importance to enhance the durability of asphalt pavement. This study aims to determine the relations between micro-mechanics, chemical composition, and macro-mechanical behavior of aged bitumen. To this end, the effect of aging on micro-mechanics, chemical functional groups, and rheological properties of bitumen were evaluated by atomic force microscope, Fourier transform infrared spectroscopy and dynamic shear rheometer tests, respectively. Results indicated that aging obviously increased the micro-surface roughness of bitumen. A more discrete distribution of micromechanics on bitumen micro-surface was noticed and its elastic behavior became more significant. Aging also resulted in raised content of carbonyl, sulfoxide, and aromatic ring functional groups. In terms of rheological behavior, the storage modulus of bitumen apparently increased after aging due to the transformation of viscous fractions to elastic fractions, making it stiffer and less viscous. By correlation analysis, it is noted that the bitumen rheological behavior was closely related to its micro-mechanics.

## 1. Introduction

Asphalt mixture, which is composed by bitumen, aggregate and mineral fillers, has been widely paved throughout the world. Compared to concrete pavement, asphalt pavement provides comfortable driving conditions with lower noise and better smoothness [1,2,3,4]. The performance of asphalt mixture is largely dependent on the rheological behavior of bitumen, which acts as a gluing binder to bond loose aggregates and fillers together. Being an organic polymer, bitumen is susceptible to aging during long-term exposure to oxygen, UV radiation and vehicle loading [5,6]. The influence of bitumen ageing on the overall performance of asphalt pavement has been well studied. It is known that aging leads to a more brittle behavior of bitumen and thus increasing the risk of fatigue cracking [7,8]. Cracks on the road surface may further accelerate bitumen aging due to the increased exposure area to air, sunlight, water and traffic loading. Thus, it is fundamental to focus on the effect of aging on chemical and rheological properties of bitumen.

Numerous studies have been conducted to investigate the effect of bitumen aging on both rheological [9,10,11] and chemical properties [12,13,14,15]. Conventionally, the aging effect of bitumen is characterized by physical and rheological parameters like penetration, ductility, softening point, Superpave rutting parameter (G*/sin δ) and phase angle [16,17,18,19]. Aged bitumen has a higher stiffness, which is beneficial to rutting resistance, but makes the binder prone to fatigue and low temperature cracking. Chemical variations refer to the formation of carbonyl compounds and sulfoxides, the transformation of generic fractions, and the increment average molecular weight and polydispersity [20,21]. Yang et al. explored the aging and rejuvenating mechanism of bitumen using Fourier Transform Infrared Spectroscopy (FTIR), proving that the aging of bitumen comes from three aspects: Loss of volatiles, dehydrogenation and oxidation [13]. Wang’s study showed that the small molecules of raw bitumen gradually transform into large molecules during the aging process and Styrene-Butadiene-Styrene (SBS) modifier degraded into small molecules with the deepening of aging [20]. Due to chemical variation, aged bitumen has higher modulus and lower phase angle, showing a solid-like rheological behavior, which is observed by increasing viscosity and brittleness as well as decreasing flexibility.

In addition to rheological and chemical characterization, several micro analysis methods were applied for mechanism investigation [22,23,24,25,26], including Scanning Electron Microscope (SEM), Transmission Electron Microscope (TEM), and Atomic Force Microscope (AFM). Especially, AFM is a technique that explores the micro-surface morphology and micro-surface mechanical properties. Loeber et al. firstly studied the microscopic surface characteristics of bitumen using AFM in 1998. A specific bee-type structure was noticed on the bitumen surface [27]. Several studies were then conducted to study the microscopic properties of bitumen, which have proven that aging significantly affects both the micro-morphology and micro-mechanics of bitumen [28,29,30,31,32,33,34,35,36,37,38,39,40,41]. For example, Wang et al. analyzed the micro-mechanical properties of five bitumen under different aging conditions using AFM. It was found that aging significantly increased the spatial variations in the sample properties and appeared to increase the sample’s adhesive and/or cohesive strength [37].

Although the effect of aging on rheological behavior and micro morphology/mechanics has been well studied, few studies focus on the correction among the aging effect on rheological behavior, chemical composition and micro-mechanics. The objective of this study, therefore, is to explore the relations between micro-mechanics, chemical composition, and macro-mechanical behavior of aged bitumen. To this end, rolling thin film oven (RTFO) and pressure aging vessel (PAV) tests were conducted for lab-simulated aging of three different types of bitumen. FTIR and Dynamic Shear Rheometer (DSR) tests were performed to investigate effect of aging on chemical composition and rheological properties, respectively. An AFM test was conducted for micro-morphology and micro-mechanical evaluation. Finally, corrections were established among results of the micro, chemical and rheological tests. This study is expected to further reveal the mechanism of bitumen aging through multi-scale characterization.

## 2. Experimental Design

### 2.1. Materials

Three typical types of bitumen with penetration grades 70 (70#), 50 (50#), and 30 (30#), supported by Shell co. ltd., was used in this study.

Bitumen samples were short-term aged with RTFO followed by long-term aged by PAV, according to AASHTO standards [42,43], Table 1 shows the physical properties of bitumen samples before and after aging.

### 2.2. Testing Program

#### 2.2.1. AFM Test

Dimension Fastscan AFM (Bruker, Santa Barbara, California, US) with peak force tapping quantitative nanomechanical (PFT QNM) mode was used to investigate the micro-morphology and micro-mechanical properties of bitumen samples. A self-designed container was developed to prepare specimens for the AFM test. The container is beneficial to ensure the consistency of the sample thickness and alleviate the substrate effect of thin samples. During the AFM test, the bitumen was oscillated in the z-direction at 1 kHz while simultaneously moving the sample in the x–y direction at a rate of 1.0 Hz (1 line/s). The PFT QNM mode adopts the Derjaguin-Muller-Toporov (DMT) model using 30% to 90% from the minimum force to the maximum peak force of every retraction curve for real-time curve fitting to obtain the DMT modulus mapping of bitumen. The accuracy of the DMT modulus measurements was ensured by controlling the sample deformation within the elastic deformation range. The topography and DMT modulus mapping were evaluated at 20 × 20 μm^2^ with 256 × 256 pixels at 20 °C.

#### 2.2.2. FTIR Test

The FTIR test was conducted to semi-quantitatively analyze the variation in the chemical compositions of bitumen before and after aging. The FTIR spectrum provides quick and reliable information about aromatic structures, aliphatic structures, and oxygenated function. The absorbance bands can be used to calculate typical functional and structural indexes [1,38,44]. In this study, an FTIR spectrometer, the VERTEX 70 (number of scans 32 and resolution 4 cm^−1^, Bruker, Ettlingen, Germany), was used to determine the functional groups of the bitumen before and after aging in a wavenumber range of 4000–400 cm^−1^. Bitumen samples for the FTIR test was firstly heated by a thermal radiative lamp. The flowable bitumen was then evenly poured onto KBr disks. The software package named Peakfit (Systat Software Inc, San Jose, CA, US) was used to analyze the spectra to obtain the area of the absorption peak.

#### 2.2.3. DSR test

A Marlvin Kinexus DSR (Marlvin, Cambridge, UK) was used to measure the rheological properties of the bitumen before and after aging. The DSR tests were conducted according to AASHTO T315. A 2-mm gap and an 8-mm-diameter plate were utilized. A strain-controlled mode was applied to perform the frequency sweep test and temperature sweep test; the applied strain of the samples was maintained within the linear viscoelastic range. To investigate the relation between the micro-mechanics and macro-mechanics, the test temperature of the temperature-sweep test was 20 °C, and the test frequency was 10 rad/s. The test temperature of the frequency-sweep test varied from −5 to 20 °C with 5 °C increments, and the test frequency varied from 0.1 to 100 Hz. The complex shear modulus (G*), storage modulus (G′) and loss modulus (G″) and phase angle (δ) were obtained from DSR analysis.

## 3. Results and Discussion

### 3.1. AFM Morphology Evaluation

**Figure 1:**
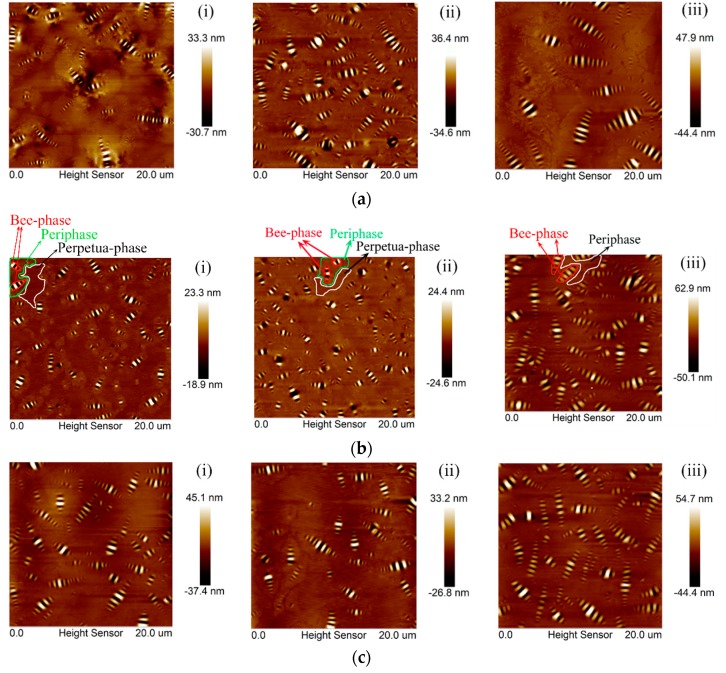
Topography of bitumen specimens before and after lab-simulated aging: (**a**) 70# bitumen; (**b**) 50# bitumen; (**c**) 30# bitumen, for (**i**) Virgin; (**ii**) rolling thin film oven (RTFO); (**iii**) pressure aging vessel (PAV).

**Figure 2:**
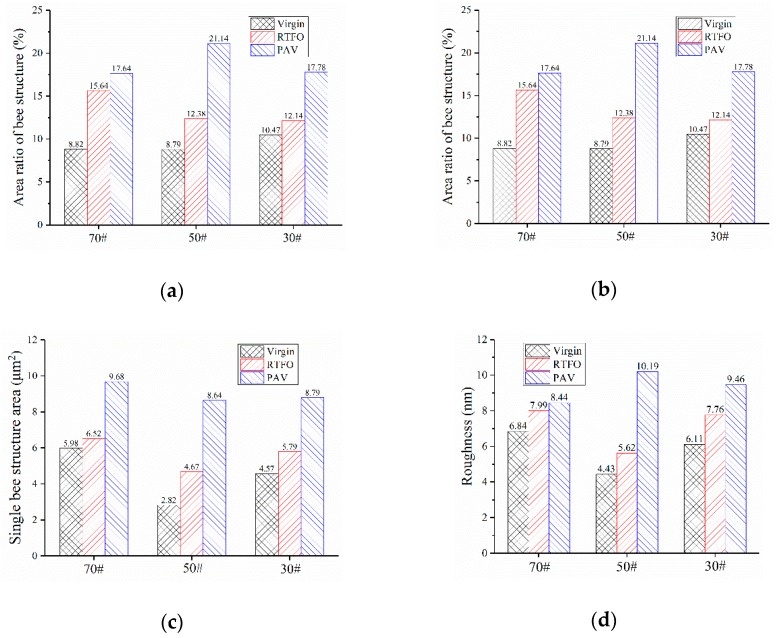
Changes in the micromorphology of bitumen before and after aging: (**a**) Bee structure number; (**b**) area ratio of bee structure; (**c**) single bee structure area; (**d**) roughness.

Figure 1 shows the typical topography of bitumen specimens before and after lab-simulated aging. The typical “bee” structure and the three-phase features (Bee phase, Periphase, and Perpetua-phase) were obviously noted in the topography (Figure 1b). According to previous studies, the bee-phase was wrapped in the Periphase and dispersed in the Perpetua-phase [41,45,46]. After aging, the bee structure became larger and the Periphase phases polymerized each other, while the Perpetua-phase almost disappeared. The boundary between the Periphase and Perpetua-phase was blurred. Those variations indicated that aging results in aggregation of bitumen surface structures, which is consistent to the bitumen colloid theory [47,48]. To quantify the effect of aging on a micro-scale level, nano-analysis was used to calculate the characteristic parameters of the bee structure and the surface roughness of the bitumen surface to analyze the variation with different aging levels. The formula for roughness calculation is shown in Equation (1) and the variation before and after aging are shown in Figure 2.

(1) $$R_{q}=\sqrt{\frac{\sum {\left(Z_{i}\right)}^{2}}{N}},$$

where Z_i_ is the current height value, and N is the number of points within the scan area.

According to Figure 2a, for both 50# and 30# bitumen, the number of bees decreased with aging, while that of 70# bitumen increased after RTFO but decreased after PAV aging. The formation of bee structure was possibility attributed to the crystallization of wax with asphaltene or other macromolecules [28,29,30,49]. Aging resulted in constant flocculation of the asphaltene, leading to fewer bee structures in both 50# and 30# bitumen. The abnormal case of 70# asphalt was probably ascribed to the relatively low content of asphaltene in the unaged stage, which was not enough to flocculate after RTFO aging.

Indicated by Figure 2b, aging significantly increased the area ratio of bee structure. After the RTFO process, the area ratio of the bee structure of the 70#, 50#, and 30# bitumen increased by 77.3%, 40.8%, and 22.2%, respectively. The increment further raised to 99.5%, 140.5%, and 70.6% for 70#, 50#, and 30# bitumen. In addition to the area ratio, the average area of the bee structure increased significantly after aging (Figure 2c). This is because of the flocculation of the asphaltene resulting from aging. Specifically, asphatltene is flocculated during aging followed by nucleating, and then the average area of the bee structure increases. The decrease in bee structure numbers and the increase in the average area promoted the increase in the area ratio of bee structure. As shown in Figure 2d, the roughness of the bitumen’s micro-surface increased as the aging degree increased, indicating that the change in the micro-structure resulted in a roughening of the asphalt micro-surface.

### 3.2. AFM DMT Analysis

**Figure 3:**
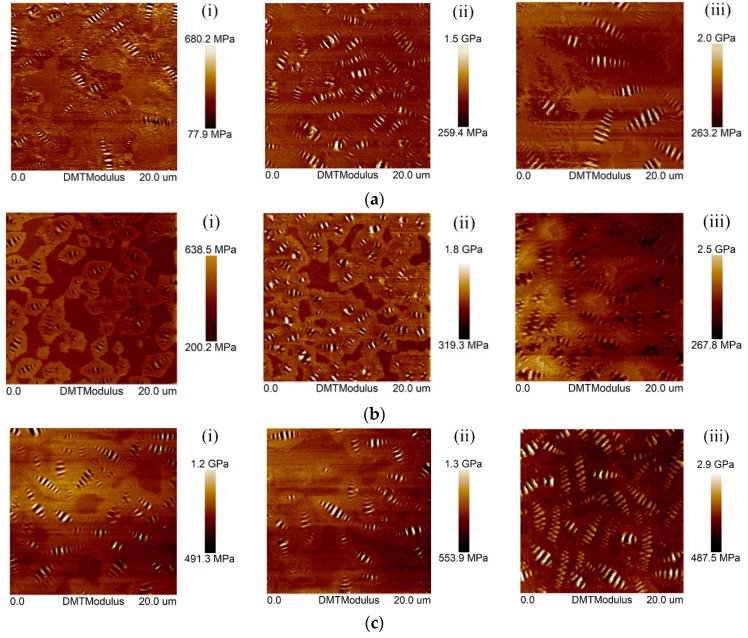
Derjaguin-Muller-Toporov (DMT) modulus of bitumen specimens before and after aging: (**a**) 70# bitumen; (**b**) 50# bitumen; (**c**) 30# bitumen, for (**i**) Virgin; (**ii**) RTFO-aged; (**iii**) PAV-aged.

**Figure 4:**
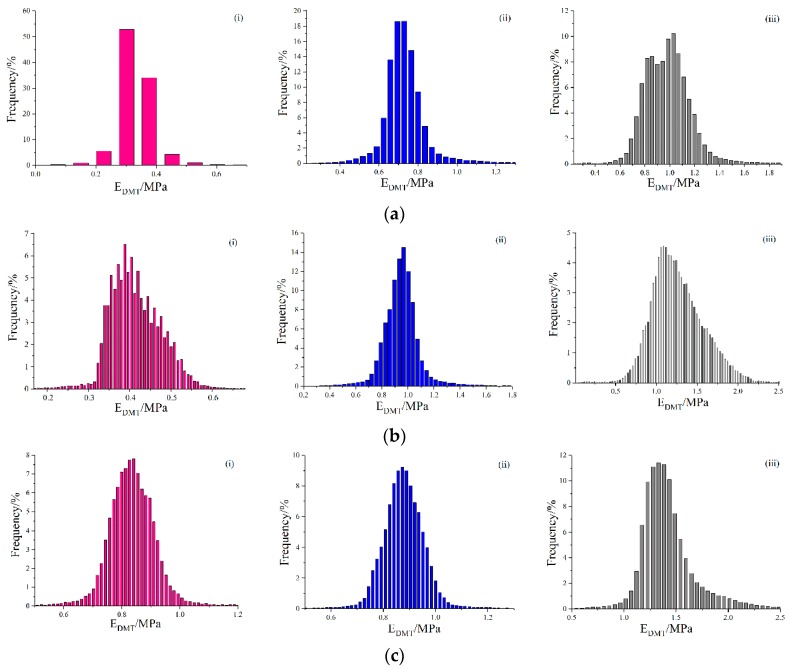
Histograms of the DMT modulus of bitumen before and after lab-simulated aging: (**a**) 70# bitumen; (**b**) 50# bitumen; (**c**) 30# bitumen, for (**i**) Virgin; (**ii**) RTFO; (**iii**) PAV.

Figure 3 shows the DMT modulus image of the selected bitumen samples and Figure 4 shows the distribution of the DMT modulus of bitumen specimens. By comparing the morphology image to the DMT modulus image, it was observed that both of them have a similar pattern, i.e., the Bee-phase, Periphase, and Perpetua-phase have the same location in both images. This indicated that the topography of the bitumen has a correlation with the distribution of the DMT modulus. The structural similarity index (SSIM) index was used for measuring the similarity between two images [38,50]. Open Source Computer Vision Library (OpenCV) were used to analyze the SSIM between the topography and the DMT modulus image of bitumen. The mean value (MV) and standard deviation (SD) of SSIM values between the topographic image and the DMT modulus image of different bitumen are shown in Table 2. It is noted that the MV of SSIM was higher than 0.8 and the SD of SSIM was less than 0.05, which demonstrated that there is a significant correlation between bitumen’s micro-surface morphology and its micro-mechanical properties. Meanwhile, Table 2 shows that the SSIM values of different bitumen were almost the same, and there was no obvious variation of MV and SD of SSIM before and after aging. This indicated that the SSIM values were independent to aging and the penetration grades of bitumen. In other words, the influence of aging and penetration grades of bitumen on SSIM values was very limited. One possible explanation for the above results is that both the microstructure and micromechanical properties of the bitumen are governed by its chemical composition. Specifically, different chemical components form different microstructures, and the morphology and distribution of the micro-mechanical properties of bitumen is dependent on its chemical compositions.

Based on Figure 4, aging led to obviously increased DMT modulus. For example, the unaged 30# bitumen had a DMT modulus of 500–1200 MPa, the modulus value increased to 700–1200 MPa after RTFO aging, and further raised to 1000–2000 MPa after PAV aging. Moreover, the distribution of the DMT modulus became more extensive.

Table 3 shows that the MV of the DMT modulus of bitumen samples. It is noted that the MV of 70#, 50# and 30# bitumen increased by 244.6%, 197.4%, and 79.7% after PAV aging, respectively. It indicated that PAV aging effectively promotes a transformation of viscous components to elastic components. Meanwhile, the SD of the DMT modulus of the 70#, 50#, and 30# bitumen increased after PAV aging by 136.4%, 288.2%, and 302.4%. These variations indicated that the aggregation of the micro-structure of bitumen surface resulted in a difference in the mechanical properties that was more significant, causing the bitumen surface to be more prone to stress concentrations when subjected to external forces.

### 3.3. Fourier Transform Infrared Analysis

**Figure 5:**
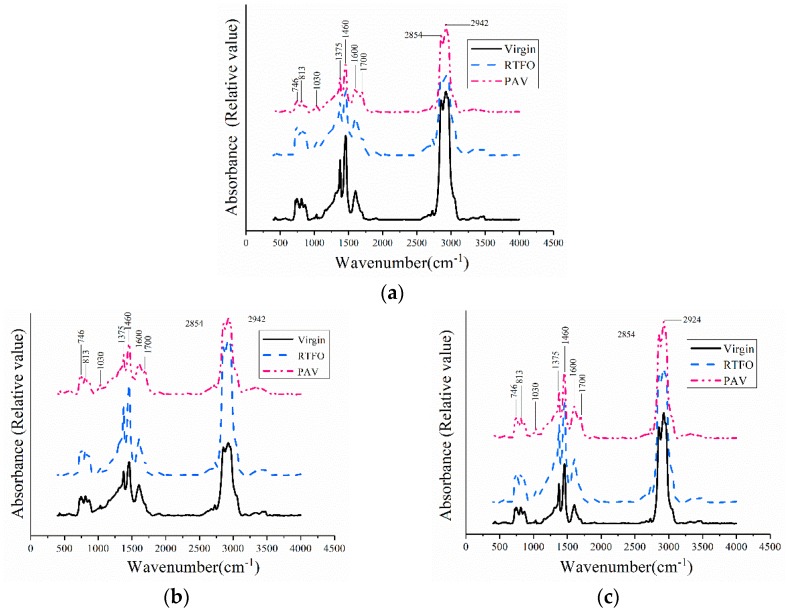
Fourier Transform Infrared Spectroscopy (FTIR) spectra of different bitumen before and after aging: (**a**) 70# bitumen; (**b**) 50# bitumen; (**c**) 30# bitumen.

FTIR spectroscopy was used to semi-quantitatively analyze the chemical compositions of bitumen in different aging levels (Figure 5). Based on Figure 5a, b and c, peaks at 1030 cm^−1^ (stretch vibration of the sulfoxide group) and 1600 cm^−1^ (C=C stretch vibration in an aromatic) were observed before and after aging. However, the peak at 1700 cm^−1^ (stretch vibration of the carbonyl group) was only observed in the FTIR spectrum of the PAV-aged samples [51]. The strong polarity of carbonyl and sulfoxide allowed a promoted intermolecular association and increased interaction force, leading to bitumen hardening and higher DMT modulus [38,52]. It is interesting to know that since the aromatic rings cannot rotate internally, the flexibility of polymer chains is reduced, which also promotes the increase of rigidity and the decrease of plasticity of polymer chains. Once the number of aromatic rings increased by aging, the modulus of bitumen will increase.

In addition to the spectrum observation, the effect of aging was evaluated by calculating the area of three typical peaks: The carbonyl functions C=O, the sulfoxide functions S=O, and the C=C stretch vibration of aromatic rings. The variation in the chemical functional groups of bitumen before and after lab-simulated aging could be obtained by calculating the indexes of the chemical functional groups [36], as shown in the following equations. According to Figure 5, the indexes of the chemical functional groups are shown in Table 4.

(2) $$I_{C=O}=\frac{Area\;of\;the\;carbonyl\;centered\;around\;1700\;m^{-1}}{\sum Area\;of\;the\;spectral\;bonds\;between\;2000\;and\;600\;cm^{-1}},$$

(3) $$I_{S=O}=\frac{Area\;of\;the\;sulphoxide\;centered\;around\;1030\;cm^{-1}}{\sum Area\;of\;the\;spectral\;bonds\;between\;2000\;and\;600\;cm^{-1}},$$

(4) $$I_{\mathrm{Aromaticity}}=\frac{Area\;of\;the\;aromatic\;ring\;centered\;around\;1600\;cm^{-1}}{\sum Area\;of\;the\;spetral\;bonds\;between\;2000\;and\;600\;cm^{-1}},$$

According to Table 4, the I_C=O_ and I_S=O_ increased obviously (2–3 times), while the I_Aromatics_ increased by approximately 10%. Due to the unstable behavior of unsaturated functional groups, during the aging process, they were very prone to oxidation, dehydrogenation, and crosslinking reactions, leading to increasing amount of carbonyl and sulfoxide functions [36,53,54]. The correlation between the DMT modulus and the chemical functional groups is shown in Table 5. It is noted that the DMT modulus was well correlated with the chemical functional groups.

**Figure 6:**
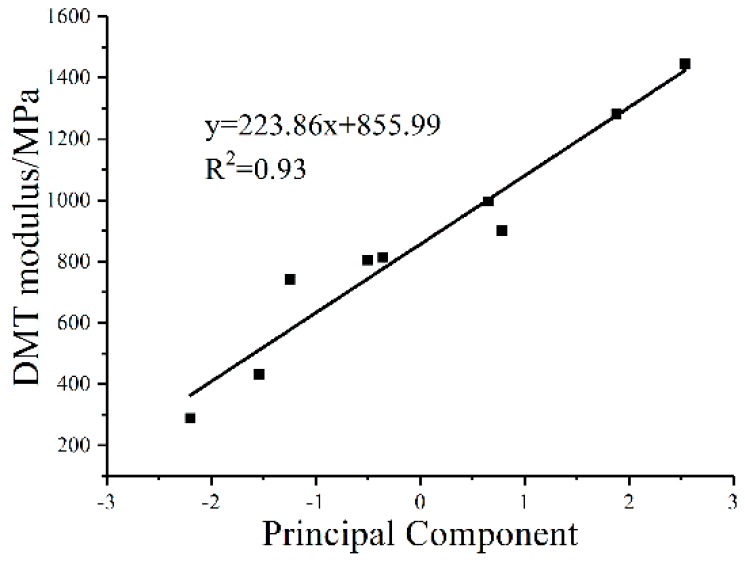
Relationship between the principal component and DMT modulus.

Regression analysis was used to investigate the synergistic effect of the chemical functional groups on the DMT modulus. The results of the Kaiser-Meyer-Olkin (KMO) and Bartlett tests are shown in Table 6. The KMO measurement of sampling adequacy is 0.56, indicating that it is suitable for factor analysis. The Sig of the Bartlett’s sphericity test is 0.00, less than the significance level of 0.01, showing that there is a correlation between the indexes of the chemical functional groups. The principal components of the chemical functional groups of bitumen before and after aging are shown in Table 7. For better illustration, regression analyses of the principal components and DMT modulus were conducted to investigate the effect of the chemical composition on microscopic properties (Figure 6). It is noted that the DMT modulus increased with an increasing principal component of the chemical functional group, and they have a good linear correlation. The results indicated that the chemical functional groups of bitumen exert a synergistic effect on the DMT modulus, and the relationship between the chemical composition of the bitumen and the micro-mechanics can be established.

### 3.4. Dynamic Rheological Characterization

**Figure 7:**
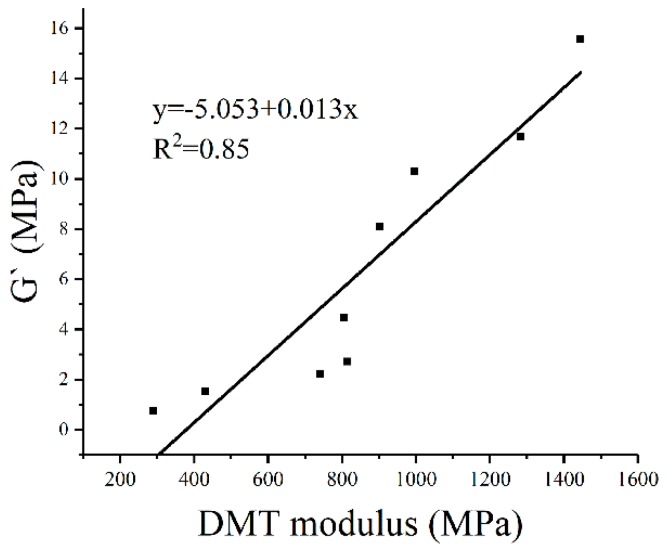
Relationship between the DMT modulus and G’.

The dynamic rheological test was performed at 20 °C (the same as the AFM test). Table 8 shows the results of complex modulus (G*), phase angle (δ) of testing bitumen. According to rheological analysis, aging significantly improved the bitumen modulus, leading to superior resistance to external loading. Besides, the phase angle (δ) decreased after aging, showing that the aged bitumen had more elastic fractions compared to the unaged sample. To establish correlation between micro-mechanics and rheological properties, the relationship between storage modulus (G’, reflecting the elastic behavior of bitumen) and DMT modulus was studied (Figure 7). It is noted that G′ increased with the DMT modulus in a general linear pattern, with a correlation coefficient R^2^ of 0.85.

**Figure 8:**
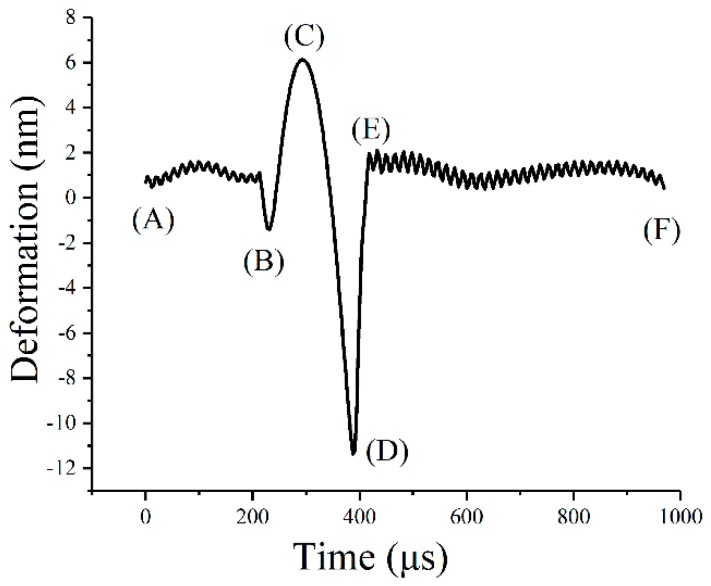
Force curve of bitumen in the Atomic Force Microscope (AFM) test.

**Figure 9:**
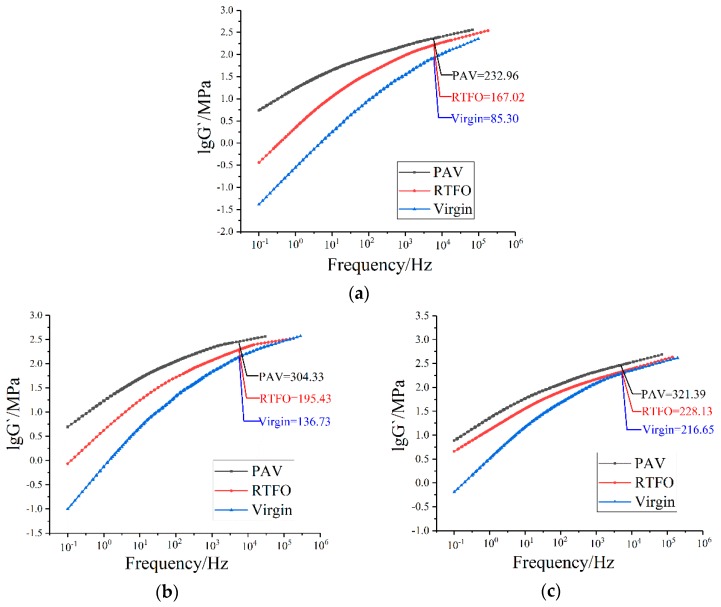
Master curves of bitumen storage modulus G′ at 20 °C: (**a**) 70# bitumen; (**b**) 50# bitumen; (**c**) 30# bitumen.

Figure 7 shows that the macro-mechanics were directly influenced by the micro-mechanics. For a more accurate correlation, the dynamic rheological analysis of bitumen was attempted to be conducted at the AFM testing frequency. Although the vibration frequency of the AFM probe is 1 kHz, the actual interaction period between the probe and the sample (from point B to point D) is only 160 μs (Figure 8). The data presented in Figure 8 illustrate the deformation variation of bitumen specimen during the whole testing process at Peakforce QNM mode: Testing start (point A), the peak of probe touches the bitumen surface (point B), the probe moves up (point B to point C), the probe goes down (point C to point D), and the probe returns back to the initial position (point D to point E). By calculation, the testing frequency of AFM is approximately 6250 Hz, which is too high to be achieved by DSR analysis. In this case, a master curve of G′ at 20 °C was constructed with test data in different temperature based on frequency-temperature superposition principle (Figure 9).

Figure 9 shows that the storage modulus G′ of testing bitumen greatly increased at a high testing frequency. By comparison, a noticeable gap is found between the modulus values by DSR and AFM analysis. This is because AFM uses the dynamic compression mode to determine the DMT modulus, while the dynamic shear mode is used in DSR. Bitumen is considered as elastic material when sustaining high frequency loading, following Equation (5):

E = 2G(1 + μ),(5)

where E, G, and μ are the compression modulus, shear modulus, and Poisson’s ratio, respectively. According to previous studies [29,34], the u value of 30# and 70# bitumen was selected as 0.4 while for 70# the u value is 0.5. By Equation 5, the macro-compression modulus values of E can be calculated (Table 9).

**Figure 10:**
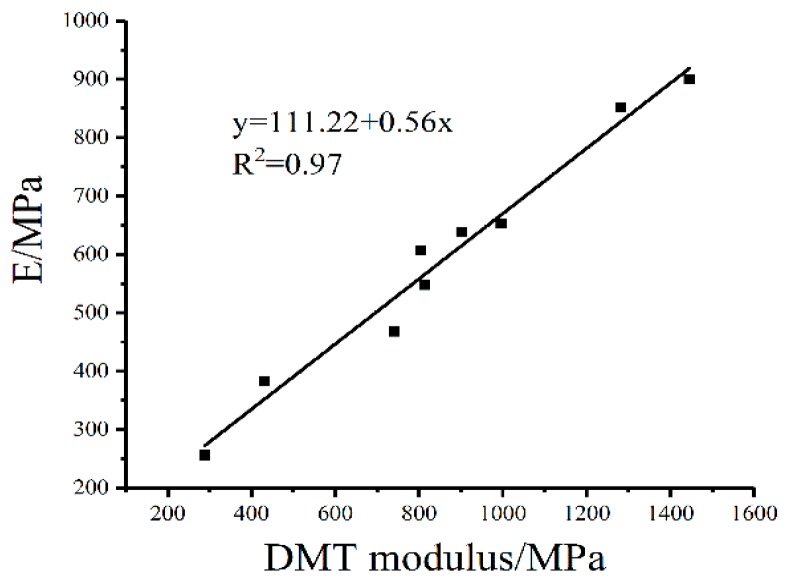
Relationship between the DMT modulus and E.

According to Table 9, the DSR macro-compression modulus values were close to the AFM DMT modulus. The macro-mechanical properties were quantitatively consistent with the micro-mechanics when the effects of testing frequency and the mode of loading were eliminated. To explore the relationship between the micro-mechanics of bitumen surface and the overall rheological properties, a regression analysis between the DMT modulus and compression modulus E was conducted (Figure 10). It is noticed that E generally increased with the DMT modulus in a linear pattern (R^2^ = 0.97), which indicated that the macro-mechanical properties are well correlated with the micro-mechanics.

## 4. Conclusions

This paper presents a comprehensive laboratory study to characterize the effect of aging on bitumen polymer by both microscopic, chemical and rheological analysis. Atomic force microscope, Fourier transform infrared spectroscopy and dynamic shear rheometer tests were performed on three types of bitumen with different penetration grades. The correlations among chemical compositions, macro-and micro-mechanics were established. Based on the experimental results and analysis, the following findings were obtained:

● Consistent to the colloid theory of bitumen, aging results in aggregation of bitumen surface and higher surface roughness.
● Aging greatly increases the DMT modulus, which results in a hardening of bitumen, making it more prone to stress concentrations when subjected to external forces.
● Aging leads to increased carbonyl, sulfoxide and aromatic ring indexes. The micro-mechanics of the bitumen surface are influenced by a synergistic effect of the chemical composition.
● Macro-mechanics is directly influenced by the micro-mechanics, and it is the embodiment of the micro-mechanics at the macro level.

This study also proven that AFM is an effective method to study both the morphology and micro-mechanics of bitumen polymer. It is able to predict the overall mechanical properties of bitumen and provide a scientific reference to analyze the mechanical behavior. Future study is recommended on applying other novel techniques to study the aging mechanism of different types of bitumen.

## Figures and Tables

**Table 1:** Physical properties of bitumen before and after aging.

Sample		Penetration (25 °C, 0.1 mm)	Softening Point (°C)	Ductility (10 °C, cm)
70#	Virgin	61	50.4	20.5
	RTFO	52	55.8	13.1
	PAV	27	63.5	1.2
50#	Virgin	55	53.1	14.7
	RTFO	34	59.7	5.9
	PAV	23	67.2	1.1
30#	Virgin	36	61.3	6.2
	RTFO	30	66.1	4.3
	PAV	13	72.9	0.3

**Table 2:** Structural similarity index (SSIM) values between the topographic image and the DMT modulus image of different bitumen before and after aging.

Sample		70#	50#	30#
Virgin	MV	0.8521	0.8946	0.8295
	SD	0.0099	0.0158	0.0294
RTFO	MV	0.9000	0.9055	0.8353
	SD	0.0105	0.0057	0.0254
PAV	MV	0.8954	0.8588	0.8611
	SD	0.0221	0.0427	0.0125

**Table 3:** Mean value (MV) and standard deviation (SD) of the DMT modulus.

Sample	70#		50#		30#	
	MV/MPa	SD/MPa	MV/MPa	SD/MPa	MV/MPa	SD/MPa
Virgin	289.47	86.15	431.21	56.37	804.67	67.03
RTFO	741.52	104.83	813.33	186.33	901.33	96.33
PAV	996.39	203.67	1281.67	218.82	1445.67	269.76

**Table 4:** Indexes of chemical functional groups.

Samples		I_C=O_	I_S=O_	I_Aromatics_
70#	Virgin	0.0379	0.0051	0.0597
	RTFO	0.0502	0.0073	0.0617
	PAV	0.0742	0.0115	0.0665
50#	Virgin	0.0467	0.004	0.0651
	RTFO	0.0618	0.0065	0.0682
	PAV	0.0917	0.0111	0.0739
30#	Virgin	0.0472	0.0064	0.0707
	RTFO	0.0725	0.0097	0.0709
	PAV	0.1045	0.0145	0.0714

**Table 5:** Correlation analysis.

Correlation Analysis	DMT Modulus		I_C=O_		I_S=O_		I_Aromaticity_	
	R^2^	Sig	R^2^	Sig	R^2^	Sig	R^2^	Sig
DMT modulus	—	—	0.95	0.00	0.92	0.00	0.79	0.01
I_C=O_	0.95	0.00	—	—	0.94	0.00	0.73	0.03
I_S=O_	0.92	0.00	0.94	0.00	—	—	0.59	0.09
I_Aromatics_	0.79	0.01	0.73	0.03	0.59	0.09	—	—

**Table 6:** Kaiser-Meyer-Olkin and Bartlett test results.

Kaiser-Meyer-Olkin Measure of Sampling Adequacy		0.56
Bartlett’s Test of Sphericity	Approx. Chi-Square	18.72
	df	3
	Sig	0.00

**Table 7:** Principal components of the chemical functional groups of the bitumen.

Samples		Component Value
70#	Virgin	−2.200
	RTFO	−1.246
	PAV	0.657
50#	Virgin	−1.542
	RTFO	−0.358
	PAV	1.877
30#	Virgin	−0.504
	RTFO	0.783
	PAV	2.532

**Table 8:** Viscoelastic properties of asphalt before and after aging at 20 °C.

Samples		G*/MPa	δ/°	G′/MPa
70#	Virgin	1.78	64.7	0.76
	RTFO	3.90	55.3	2.22
	PAV	13.37	39.7	10.29
50#	Virgin	2.99	59.4	1.52
	RTFO	4.57	53.5	2.72
	PAV	14.60	36.8	11.69
30#	Virgin	6.69	48.1	4.47
	RTFO	10.50	39.6	8.09
	PAV	17.76	28.8	15.56

**Table 9:** Macro-compression modulus E of bitumen before and after aging tested at 6250 Hz.

Samples		E/MPa	DMT Modulus/MPa
70#	Virgin	255.91	289.47
	RTFO	467.65	741.52
	PAV	652.29	996.39
50#	Virgin	382.84	431.21
	RTFO	547.2	813.33
	PAV	852.13	1281.67
30#	Virgin	606.61	804.67
	RTFO	638.75	901.33
	PAV	899.89	1445.67
